# Integration between Primary Care and Mental Health Services in Italy: Determinants of Referral and Stepped Care

**DOI:** 10.1155/2012/507464

**Published:** 2012-05-20

**Authors:** Paola Rucci, Antonella Piazza, Marco Menchetti, Domenico Berardi, Angelo Fioritti, Stefano Mimmi, Maria Pia Fantini

**Affiliations:** ^1^Dipartimento di Medicina e Sanità Pubblica, Alma Mater Studiorum Università di Bologna, Via San Giacomo 12, 40124 Bologna, Italy; ^2^Dipartimento Salute Mentale e Dipendenze Patologiche, Azienda USL di Bologna, Viale Pepoli 5, 40123 Bologna, Italy; ^3^Istituto di Psichiatria, Alma Mater Studiorum Università di Bologna, Viale Pepoli 5, 40123 Bologna, Italy

## Abstract

This study, carried out in the context of a collaborative care program for common mental disorders, is aimed at identifying the predictors of Primary Care Physician (PCP) referral to Community Mental Health Center (CMHC) and patterns of care. Patients with depression or anxiety disorders who had a first contact with CMHCs between January 1, 2007–December 31, 2009 were extracted from Bologna Local Health Authority database. A classification and regression tree procedure was used to determine which combination of demographic and diagnostic variables best distinguished patients referred by PCPs and to identify predictors of patterns of care (consultation, shared care, and treatment at the CMHC) for patients referred by PCPs. Of the 8570 patients, 57.4% were referred by PCPs. Those less likely to be referred by PCPs were living in the urban area, suffered from depressive disorder, and were young. As to the pattern of care, patients living in the urban area were more likely to receive shared care compared with those living in the nonurban area, while the reverse was true for consultation. Predictors of CMHC treatment were depression and young age. Prospective studies are needed to assess length, quantity, and quality of collaborative treatment for common mental disorder delivered at any step of care.

## 1. Introduction

Mental disorders are very common in primary care setting: the WHO Collaborative Study on Psychological Problems in General Health Care (PPGHC) reported a global prevalence of 24.0% [1]. In this study, that excludes schizophrenia spectrum disorders, the most frequent disorders were Depression (10.4%) and Generalized Anxiety Disorder (7.9%). The crucial role of primary care in the recognition and the management of mental disorders is receiving growing attention, and collaboration projects between Primary Care and Mental Health sectors are underway in many countries following models developed in the United Kingdom and in the USA [2–5].

In Italy, primary care is placed at the heart of the health care system. Primary care physicians (PCPs) are independent contracted professionals who operate under the control of Local Health Authorities (LHAs).

On average, LHAs are responsible for the overall health of, and for the services offered to, a target population of 350,000 inhabitants. PCP are the first contact for the most common health problems and act as gatekeepers for drug prescription and for access to specialty and hospital care.

Specifically, PCP are involved in delivering various primary care services like health promotion and preventive care activities, diagnosis, treatment, and followup of noncomplex, acute, and chronic conditions. They also have an increasing role as coordinators of services provided to patients with chronic diseases [6]. Essential health services are provided free of charge, or at a minimal charge. The extension of universal health care coverage to the whole population is in fact a key characteristic of the Italian health care system.

Mental health care in Italy is currently delivered by Mental Health Departments (MHDs) that are in charge of the management and planning of all medical and social activities related to prevention, treatment, and rehabilitation in a defined catchment area. Within the departments, Community Mental Health Centers (CMHCs) cover all activities pertaining to adult psychiatry in outpatient settings and manage therapeutic and rehabilitation activities delivered by day care services and nonhospital residential facilities [7]. Public in-patient care is provided by general hospital psychiatric units, university psychiatric clinics, and community mental health centers operating 24 h a day [8]. 

Among the existing attempts to promote the integration between primary care and mental health services, the “G. Leggieri” Program was started in 2000 as an effort of the Health Government of Emilia-Romagna Region to coordinate initiatives of primary care-mental health cooperation undertaken since the 1980's. A steering group of representatives of local health authorities, mental health and primary care departments, scientific associations, and academic institutions was established in order to deliver specific recommendations about the organisation of collaborative activities. In particular, two main objectives were identified and pursued: (1) to improve the quality of treatment for patients with common psychiatric disorders in primary care; (2) to modify the pathways of care, supporting the management of common psychiatric disorders in primary care and focusing mental health services' activities towards severe or difficult-to-treat cases.

In the framework of this program, regional recommendations were delivered according to the stepped care model proposed in the NICE guidelines for depression and anxiety [9, 10]. Stepped care is a system of health care based on treatments of differing intensity graded to the patient's needs, so that the least intrusive or restrictive intervention is first provided. For the management of common mental disorders five steps were devised: PCP treatment, PCP treatment supervised by CMHC, consultation with CMHC, shared care between PCP and CMHC, and treatment at the CMHC. These steps are ordered along a dimension of increasing CMHC involvement and decreasing PCP responsibility; referral of patients to CMHC is envisaged from the third step upwards.

Within this regional context, local models of collaborative care have been gradually refined [11–13]. This paper deals with the experience carried out at the LHA of Bologna, an urban-rural catchment area including the metropolitan area of Bologna.

The specific aims of present study are (1) to determine in which proportion patients with common mental disorders who have a first contact with the CMHCs are referred by PCPs; (2) to identify the predictors of PCP referral versus other referral sources; (3) to predict the pattern of care of patients referred by PCPs, as a function of their diagnosis and demographic characteristics.

## 2. Materials and Methods

### 2.1. Setting and Data Source

The data source for this study is the mental health information system of the Bologna LHA. The Bologna LHA is one of the largest in the country and serves approximately 850,000 inhabitants, roughly one fifth of the regional population.

The mental health information system was implemented in 2007 for administrative and clinical-epidemiological purposes. All patients who had at least one contact with community-based mental health services were recorded in the database since then, reflecting the total secondary mental health care in the area. Data include, in addition to patient's ID number, demographic characteristics, the patient's PCP name, the ICD-9 CM diagnosis, information on each type of intervention administered, and the number and type of staff involved in the intervention.

### 2.2. Study Sample

Demographic and diagnostic information of patients who had their first contact with one of the 11 CMHCs of the LHA between January 2007 and December 2009 was extracted from the database. Patients who were not living in Bologna province, those with a missing diagnosis or with fewer than 18 years of age were excluded from the analyses.

ICD-9 CM diagnoses were classified into 9 groups: schizophrenia (295, 297, 298 excl. 298.0, 299), depression (296.2-3, 296.9, 298.0, 300.4, 309.0, 309.1, 311), bipolar disorders (296.0, 296.1, 296.4–8), personality disorders (301, 302, 312), alcohol and substance use disorders (291, 292, 303, 304, 305), dementia and organic mental disorders (290, 293, 294, 310), anxiety and somatoform disorders (300 (excl. 300.4), 306, 307.4, 307.8-307.9, 308, 316), mental retardation (317, 318, 319), and other mental disorders (307.0–307.3, 307.5–307.7, 309.2–309.9, 313, 314, 315).

Patients with depression or anxiety and somatoform disorders, who are the target of Leggieri Program, were retained for the classification tree analyses.

### 2.3. Patterns of Care

As mentioned above, there are 3 steps that entail direct CMHC involvement after referral by PCP.

#### 2.3.1. Consultation

This includes the establishment of a psychiatric diagnosis, significant life events, and the description of possible dysfunctional coping behaviors. The evaluation is followed by suggestions for the treatment plan, which is delivered by the PCP. Information is then forwarded to the PCP in a typed report designed to be thorough, but concise.

#### 2.3.2. Shared Care

After the assessment, the consultant provides brief and focused therapeutic interventions to support the PCP management of psychiatric disorders. Written communications are accompanied by telephone communication or interpersonal contacts in order to increase understanding and cooperation between psychiatrists and PCPs. For example, the psychiatrist could start pharmacological treatment and furthermore evaluate the initial treatment response and patient compliance. In other cases, a brief psychological intervention can be provided (counseling).

#### 2.3.3. Treatment at the CMHC

Severe and complex cases are referred to the CMHC that takes full responsibility for psychiatric management.

### 2.4. Statistical Analyses

In this study, patients referred to the CMHCs of Bologna area with a diagnosis of anxiety or depressive disorder were classified into two mutually exclusive groups according to the referral source (PCPs versus other sources).

To determine which combination of demographic and diagnostic variables best distinguished patients referred by primary care physicians from those referred by other sources, data were analyzed using a classification and regression tree (CART) procedure.

This procedure was also used to determine which characteristics (and combination of characteristics) predicted the pattern of care of patients referred by primary care physician (consultation, shared care, and treatment at the CMHC).

In contrast to traditional statistical models, CART is a nonparametric analysis that simultaneously examines interactions between continuous or categorical variables to create a decision tree. Researcher bias is limited as CART can use large numbers of variables to create a decision tree and cutoff on continuous variables that are defined by the procedures.

To date, there are no studies that employed these methods to predict outcomes such as referral to PCP or patterns of care in patients referred to CMHC, but this method has been recently used in psychiatry [14, 15] and other medical fields, including for instance cardiology and nephrology [16, 17] Because of its ability to identify significant interactions, it has been used to analyse the influence of gene-environment interactions in lung cancer [18].

The CART procedures build decision trees beginning with a root node that includes all cases, then the tree branches into two subgroups (or nodes) and grows iteratively by identifying optimal cut points for continuous discriminating variables in the predictor set. Categories of nominal variables (such as diagnosis or marital status) are merged by the procedure if the distribution of the dependent variable is similar across the categories. The best discriminating predictor is selected first, and then subsequent predictors are entered into the procedure if they contribute significantly to subtyping cases into homogeneous groups. Variables not useful to discriminate cases do not enter into the procedure. The tree grows until a stopping criterion is met or no further significant improvement in the classification of study participants is possible. At the end of the procedure, the study population is partitioned into terminal nodes that are as homogeneous as possible with respect to the categories of the dependent variable. The final tree is “pruned” to avoid model overfitting. This is done by a procedure that, after the tree is grown in its full depth, trims it down to a smaller subtree that has an acceptable risk of misclassification (defined as 1 standard error with respect to the risk in the full tree). All analyses were carried out using SPSS, version 17.0 (Chicago, IL).

## 3. Results and Discussion

### 3.1. First Tree: Predictors of PCP Referral

8570 patients with depression or anxiety and somatoform disorders, that constituted 56.4% of first contacts at the CMHC were included in the classification tree analysis. The proportion of patients with these disorders referred by primary care physicians to the CMHCs was 57.4%.

The proportion of PCP referrals increased from 51.5% to 62.2% in the period 2007–2009.

The classification tree analysis included as independent variables diagnosis, age, gender, educational level, marital status, nationality (Italian versus other), and area of residence (urban versus nonurban).

**Figure 1:**
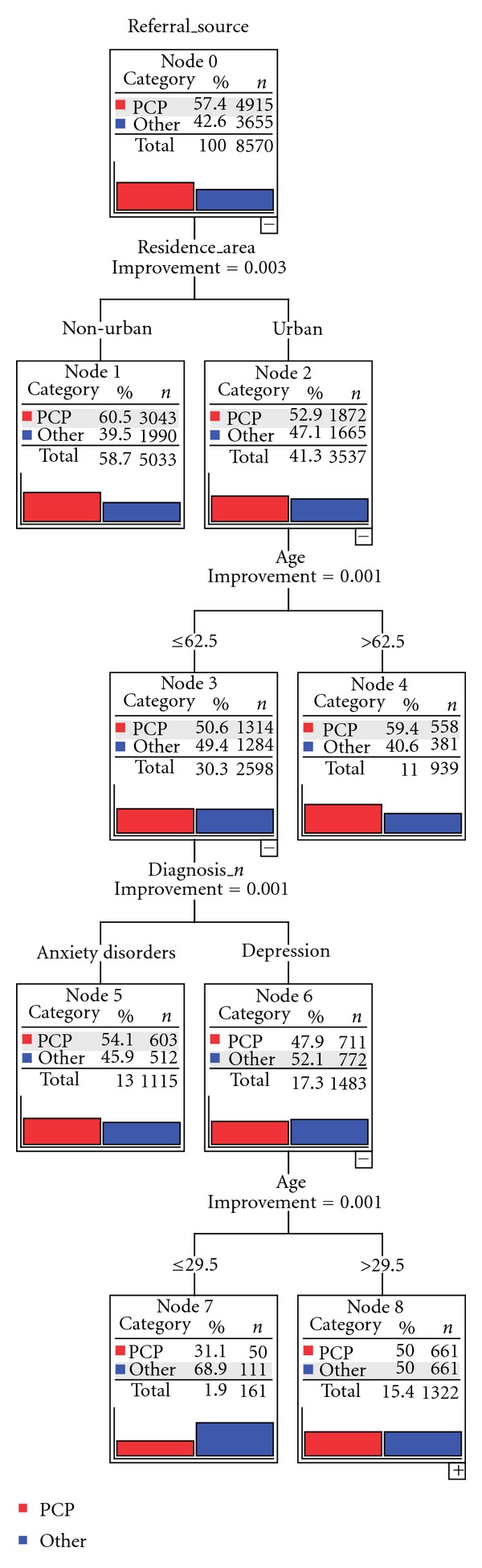
Classification tree analysis showing the predictors of PCP referral versus other referral sources.

Results (Figure 1) indicate that patients living in a nonurban area (node 1) were more likely to be referred by PCP than those living in the urban area (node 2, 60.5% versus 52.9%). In the latter subgroup, patients were further split according to an age cutoff of 63 years, that separated those who were more likely to be referred by PCPs compared to younger patients (59.4% versus 50.6%). In patients with an age <63 years, a diagnosis of anxiety disorder (compared to depressive disorder) proved to be a significant predictor of PCP referral (54.1% versus 47.9%). Among patients with depression (node 6), another split was related to age. In fact depressed patients with an age >30 years were more likely to be referred by PCP compared to younger patients (50% versus 31.1%).

In summary, among patients with common mental disorders in contact with CMHCs, those less likely to be referred by PCPs were living in the city, suffered from a depressive disorder, and were young. This suggests that young urban depressed patients could face barriers in help-seeking for a variety of factors. As widely recognized [19, 20], the young and the depressed have unmet needs of care, most often in urban environments, and this deserves greater attention in next phases of the Leggieri Program implementation.

### 3.2. Second Tree: Predictors of Pattern of Care

Of the 4913 patients referred by PCPs, 1276 (26%) received a consultation, for 765 (15.6%) shared care was agreed between the CHMC and the PCP and for the remaining 2872 (58.5%) the CMHC was exclusively in charge of the intervention. A strong increase in the shared care pattern was observed from 2007 to 2009, paralleled by the decrease of treatment at the CMHC (Table 1).

**Figure 2:**
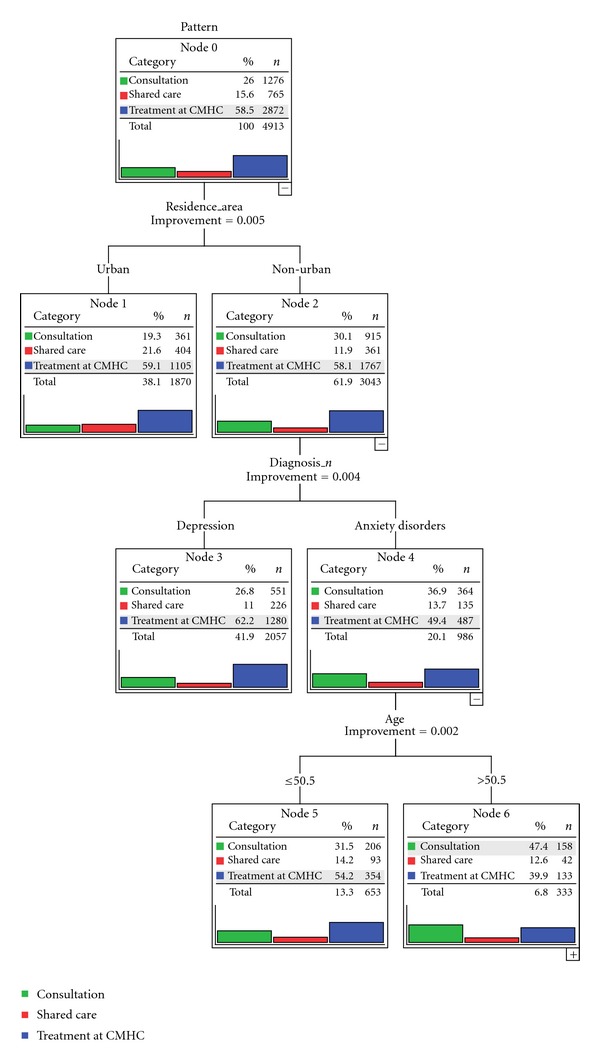
Classification tree showing the predictors of pattern of care among patients referred by PCPs.

The classification tree analysis (Figure 2), using the same independent variables as in the first tree, indicated that residence area was again the most important discriminator of pattern of care. Patients living in the urban area were more likely to receive shared care compared with those living the nonurban area (21.6% versus 11.9%), while the reverse was true for consultation (19.3% versus 30.1%). On the contrary, the proportion of patients treated at the CMHC was similar between urban and nonurban areas. Still, in the subgroup of patients living in nonurban areas, there was a differential pattern of care according to the diagnosis. Depressed patients were more likely to be treated at the CMHC compared with patients with anxiety disorders (62.2% versus 49.4%). In this latter subgroup of patients with anxiety disorders and living in nonurban areas, consultation was more likely if their age was ≥50.5 years (node 6) and treatment at the CMHC more likely if their age was ≤50.5 years (node 5). 

In summary, we observed a different pattern of collaborative care in urban compared with nonurban areas. Consistent with our expectations, predictors of CMHC treatment were depression versus anxiety disorders and young age versus older age.

## 4. Conclusions

Our results should be interpreted keeping in mind that the focus is on patients with common mental disorders *referred* to CMHCs. Therefore, the present study does not allow either to estimate the prevalence of common mental disorders in primary care or to determine the proportion of patients actually treated by the PCPs. Still, the availability of the LHA mental health data offers the unique opportunity to analyze the type of care provided to patients once they are referred to the CMHC and the extent to which collaborative care between PCPs and community-based services is actually delivered.

Patients with common mental disorders (anxiety or depression) comprised 56.4% of referrals, more than half of them (57.4%) were referred by PCPs with a trend rapidly growing over time (from 51.5% to 62.2%). The pathway from PCP to CMHC appears to have strengthened in comparison to that reported in other previous Italian investigations, such as in South-Verona, where PCP referrals accounted for 40% of new cases in 2000 [21]. Since then the PCPs' gatekeeper role has been even more recognised as a priority in improving access to specialized mental health care. Multiple integration strategies could further help this evolution, like face-to-face communication, counselling in colocated accommodation, training in small groups and regular feed-back between PCP and mental health professionals. In 2007, the steering group of the “G. Leggieri” Program developed a document with recommendations for the management of common mental disorders between primary care and mental health services. Besides this initiative, the institution of a formal Primary Care Department and of “functional” primary care groups of about 15–20 PCPs made the liaison and the training more easy to organize.

The classification tree analysis suggests that the residence area plays an important role in the PCP referral process that appears to be more active in the nonurban compared to the urban area. In our opinion this finding is partially related to the organizational characteristics of Primary Care Services. In fact, urban PCPs often run individual practices, whilst nonurban PCPs are frequently associated in group practices, located in the same outpatient clinic. This organization fosters access, continuity of care, training opportunities and plays a part in the integration with mental health services. Moreover, nonurban practitioners have traditionally stronger links with their community and other health services (including mental health services), which favors their role of gatekeepers to secondary care [22].

In the urban area older patients had increasing odds to be referred to CMHCs compared with younger patients. A possible interpretation is that patients with an age of 63 years or more are probably more familiar with their PCP and more inclined to endorse their psychological complaints during the visit. Among patients <63 years of age, PCP referral was more common for anxiety than for depressive disorders. Of note, among depressed patients an age <30 years seemed to further disfavor PCP referral.

By and large, these data highlight possible barriers that young people have to face in accessing health services and their unmet needs especially in urban areas. As many authors have noted [23, 24], enough is known to recommend as a priority the provision of innovative and well assessed youth-friendly primary and mental health services.

As to the pattern of care of patients referred by PCPs, examined in the second classification tree, we found that shared care was more frequent in urban than in nonurban areas, while consultation was more frequent in nonurban areas. Increasing either consultation or shared care was a targeted priority of the Leggieri Program, but in both areas a nearly 60% of common mental disorders referred by PCPs still receive specialized treatment at the CMHC, although this percentage was decreasing over the investigated three-year period (from 71.5% in 2007 to 44.8% in 2009). However, in the next future we expect a further decrease of this pattern of care for common mental disorders, as a result of increasing cooperation and PCP training.

It is remarkable that other demographic characteristics such as gender, marital status, educational level, and nationality, that traditionally play a role in the help-seeking behaviors and in service utilization patterns [25], did not emerge as predictors, neither for type of referral nor for pattern of care.

Overall, our findings provide a preliminary evidence on the implementation of the Leggieri Program in Bologna LHA. Future perspectives include the design of policies aimed at removing barriers in accessing mental health services for young people, mainly in the urban environment. Moreover, prospective studies using the Bologna LHA mental health database are needed to assess length, quantity, and quality of treatment delivered at any step of care in order to ensure that patients with common mental disorders receive appropriate and effective integrated care.

## Figures and Tables

**Table 1:** Pattern of care distribution over time in patients referred by PCPs.

			Year of first contact			Total
			2007	2008	2009	
Pattern	Consultation	*N*	360	384	532	1276
		%	24.9%	22.3%	30.5%	26.0%
	Shared care	*N*	52	281	432	765
		%	3.6%	16.3%	24.7%	15.6%
	Treatment at CMHC	*N*	1033	1056	783	2872
		%	71.5%	61.4%	44.8%	58.5%

Total		*N*	1445	1721	1747	4913
		%	100.0%	100.0%	100.0%	100.0%
